# Autophagy Stimulus Promotes Early HuR Protein Activation and p62/SQSTM1 Protein Synthesis in ARPE-19 Cells by Triggering Erk1/2, p38^MAPK^, and JNK Kinase Pathways

**DOI:** 10.1155/2018/4956080

**Published:** 2018-02-08

**Authors:** Nicoletta Marchesi, Natthakan Thongon, Alessia Pascale, Alessandro Provenzani, Ali Koskela, Eveliina Korhonen, Adrian Smedowski, Stefano Govoni, Anu Kauppinen, Kai Kaarniranta, Marialaura Amadio

**Affiliations:** ^1^Department of Drug Sciences, Pharmacology Section, University of Pavia, 27100 Pavia, Italy; ^2^Laboratory of Genomic Screening, Center for Integrative Biology, University of Trento, 38123 Trento, Italy; ^3^Department of Ophthalmology, University of Eastern Finland, 70211 Kuopio, Finland; ^4^School of Pharmacy, Faculty of Health Sciences, University of Eastern Finland, 70211 Kuopio, Finland; ^5^Chair and Department of Physiology, School of Medicine in Katowice, Medical University of Silesia, Katowice, Poland; ^6^Department of Ophthalmology, Kuopio University Hospital, 70029 Kuopio, Finland

## Abstract

RNA-binding protein dysregulation and altered expression of proteins involved in the autophagy/proteasome pathway play a role in many neurodegenerative disease onset/progression, including age-related macular degeneration (AMD). HuR/ELAVL1 is a master regulator of gene expression in human physiopathology. In ARPE-19 cells exposed to the proteasomal inhibitor MG132, HuR positively affects at posttranscriptional level p62 expression, a stress response gene involved in protein aggregate clearance with a role in AMD. Here, we studied the early effects of the proautophagy AICAR + MG132 cotreatment on the HuR-p62 pathway. We treated ARPE-19 cells with Erk1/2, AMPK, p38^MAPK^, PKC, and JNK kinase inhibitors in the presence of AICAR + MG132 and evaluated HuR localization/phosphorylation and p62 expression. Two-hour AICAR + MG132 induces both HuR cytoplasmic translocation and threonine phosphorylation via the Erk1/2 pathway. In these conditions, p62 mRNA is loaded on polysomes and its translation in de novo protein is favored. Additionally, for the first time, we report that JNK can phosphorylate HuR, however, without modulating its localization. Our study supports HuR's role as an upstream regulator of p62 expression in ARPE-19 cells, helps to understand better the early events in response to a proautophagy stimulus, and suggests that modulation of the autophagy-regulating kinases as potential therapeutic targets for AMD may be relevant.

## 1. Introduction

Posttranscriptional mechanisms are key determinants in the modulation of gene expression by allowing a punctual, localized adaptation of protein levels to changing environmental conditions. In particular, RNA-binding proteins (RBPs) are predicted to regulate up to 90% of human genes, and their physiological role is critical for the maintenance of health conditions in all tissues, including the eye [1–3]. Recent evidence has shown that the dysregulation of RBPs controlling the expression of proteins involved in the autophagy/proteasome pathway has a role in the onset and the progression of many neurodegenerative diseases [4].

The RBP HuR (human antigen R or HuA) is a master regulator of gene expression in several physiological and pathological conditions. HuR (also named ELAVL1 (embryonic lethal abnormal vision-like 1)) belongs to the mammalian ELAV family, one of the most abundant and the best-known RBPs affecting the RNA fate at various levels. ELAV (or Hu) proteins interact preferentially with adenine-uracil-rich elements (ARE) mainly, but not exclusively, present in the 3′-untranslated region of a high number of mRNAs [5]. Despite the high homology in the primary sequences among the four ELAV members, certain specificity for their localization, behavior, function, and target mRNAs has been evidenced [6, 7]. The so-called neuronal ELAV proteins, namely, HuB, HuC, and HuD, are almost exclusively present in neurons and mostly localized in the cytoplasm [8]. HuR is expressed in all tissues and in basal conditions remains mainly within the nucleus [9]. Following an extracellular stimulus (such as stress), HuR protein shuttles from the nucleus to the cytoplasm, where it can increase the stability and/or the rate of translation of the bound transcripts [10, 11]. HuR's targets include mRNAs coding proteins involved in the cellular stress response and survival, inflammation, and cell cycle progression [12–17]. We previously showed that under proteasome inhibition, HuR posttranscriptionally affects the expression of p62/sequestosome 1 (SQSTM1) in a retinal pigment epithelial (RPE) cell line. p62 is a key factor to regulate protein aggregate clearance via autophagy and proteasome pathways that are involved in the pathology of age-related macular degeneration (AMD) [18].

Autophagy is a stress-responsive process playing a crucial role in the homeostasis of cells and tissues, especially in the retina, where the postmitotic RPE cells are primarily responsible for the phagocytosis of photoreceptor outer segments, thereby promoting the retina's health [19, 20]. One of the early triggering factors in the pathogenesis of AMD is the degeneration of RPE. During aging, RPE cells show increased susceptibility to oxidative stress and increased protein aggregation due to impaired autophagy and proteasome-mediated proteolysis [21, 22], which finally contributes to the RPE cell death [23].

Accumulating evidence suggests that autophagy proceeds in two phases: first, within minutes or hours of exposure to a stressful condition, a rapid activation of stress proteins and protective mechanisms takes place, and it is mainly mediated by posttranslational protein modifications. After that, a delayed and sustained stress response, relying on the activation of programs modifying gene expression at the transcriptional level, occurs [24].

With the aim to dissect the early phase of autophagy induction on the HuR-p62 pathway, we exposed ARPE-19 cells to the proautophagy AICAR and MG132 cotreatment and evaluated the p62 expression and HuR activation. The list of signaling pathways directly or indirectly involved in the nucleocytoplasmic HuR shuttling and/or HuR phosphorylation (both of the indexes of HuR activation) is long [25, 26]. Therefore, we focused on those kinases affecting the cellular localization of HuR and/or its binding to target RNAs and whose relevance in the cellular stress response and/or autophagy has been acknowledged. In particular, the involvement of extracellular signal-regulated kinase [Erk1/2, also known as p-44/42 mitogen-activated protein kinase (MAPK)], AMP-activated protein kinase (AMPK), p38^MAPK^, c-Jun N-terminal kinase (JNK), and protein kinase C (PKC) was studied in ARPE-19 cells.

## 2. Materials and Methods

### 2.1. Cell Culture and Treatments

The human RPE cell line ARPE-19 was obtained from American Type Culture Collection. Cells were grown in a humidified 5% CO_2_ atmosphere at 37°C in Dulbecco's Modified Eagle Medium: F12 (1 : 1; Gibco, Invitrogen, Carlsbad, CA), including 10% inactivated fetal bovine serum, 100 units/ml penicillin, 100 *μ*g/ml streptomycin, and 2 mM L-glutamine (Sigma-Aldrich, Milan, Italy). To find out the best conditions for studying both HuR protein translocation and p62 protein expression, cells were exposed to either the solvent (DMSO, 0.1%), the proteasome inhibitor MG132 (1 *μ*M, Calbiochem, San Diego, CA), or AICAR (2 mM 5-aminoimidazole-4-carboxy amide ribonucleoside, Toronto Research Chemical, Canada), alone or together, for 15 min, 30 min, or 2 hrs. AICAR and MG132 (A + M) cotreatment for 2 hrs was selected for all the following experiments. Protein synthesis was inhibited by 1 *μ*M puromycin (Sigma-Aldrich). Kinase inhibitors were used at the concentrations suggested by the manufacturers or optimized in previous studies [27, 28]—PD98059 (MEK1/2 inhibitor; Cell Signaling, Danvers, MA): 50 *μ*M; compound C (CC, AMPK inhibitor; Sigma-Aldrich): 5 *μ*M; SB203580 (p38^MAPK^ inhibitor; Cell Signaling): 50 *μ*M; SP600125 (JNK-1,-2, and −3 inhibitor; Cell Signaling): 10 *μ*M; and Gö6976 (Ca^2+^-dependent PKC inhibitor; Calbiochem): 2 *μ*M. Each inhibitor was added to the cell culture medium at least 15 min before the A + M cotreatment and maintained until the end of the experiment.

### 2.2. LDH Experiment

To evaluate the plasma membrane damage and the cell viability at 24 hrs, a colorimetric assay for measuring lactate dehydrogenase (LDH) was performed on ARPE-19 cell culture medium samples. The medium was tested using the LDH substrate included in a commercial kit (cytoxicity detection kit, Roche, Molecular Biochemicals, Mannheim, Germany). The absorbance values were measured at 450 nm using a microplate reader (Synergy HT Multi-Mode, Bio-Tek), and results were expressed as percentages of control (100%).

### 2.3. Cell Fractioning

After exposures, cells were washed twice with cold phosphate-buffered saline (PBS), scraped, and collected. Before cellular fractioning, a small volume of cell homogenate was held and analyzed as total lysate. Nuclear and cytoplasmic extracts were separated by using the Nuclear Extract kit (Active Motif, Carlsbad, CA) according to [18].

### 2.4. Western Blotting

Proteins of whole cell lysates and nuclear and cytoplasmic fractions were separated on 10% or 12% SDS-polyacrylamide gel electrophoresis and processed following the standard procedures. Briefly, the nitrocellulose membrane was washed with 0.1% Tween20 in Tris-buffered saline (TTBS), incubated for 1 hr at the room temperature (RT) with 5% non-fat milk in TTBS (blocking solution), and incubated overnight at 4°C with the primary antibody diluted in milk–TTBS. Specific antibodies for HuR (1 : 1000), p62 (1 : 800), phosphothreonine (1 : 750), phosphoserine (1 : 750) (all from Santa Cruz Biotechnology Inc., Santa Cruz, CA), phospho-p-44/42 MAPK (Erk1/2) (Thr202/Tyr204) (Cell Signaling), beclin-1, *α*-tubulin (Sigma-Aldrich), and lamin C (Abcam, Cambridge, UK) were diluted as suggested by the manufacturers. Membranes were then washed and incubated with HRP-conjugated secondary antibodies diluted in milk–TTBS for 1 hr at RT. The immunoreactive bands were visualized by chemiluminescence. Experiments were performed in duplicate for each different cell preparation. As for loading controls, *α*-tubulin was used for both total homogenate and cytoplasm, while lamin C for the rough nuclear fraction, respectively. The same proteins were also used as purity controls for each cellular fraction; however, according to [18], *α*-tubulin was detectable also in rough nuclei when loading ~40 *μ*g of protein extract. Statistical analysis of the Western blotting data was performed on the densitometric values obtained by quantifying the immunoblots with the Scion Image software (Scion Corporation) after the image acquisition.

### 2.5. Real-Time Quantitative PCR

RNA was extracted from whole cell homogenates, cytoplasmic fractions, or immunoprecipitated samples by the RNeasy-Plus Micro Kit (Qiagen, Milan, Italy) and subjected to reverse transcription following standard procedures. Real-time quantitative PCR (qPCR) amplifications were carried out using the Lightcycler instrument (Roche), with the following primers:

HuR: 5'-GAGGCTCCAGTCAAAAACCA-3′ (upstream) and 5′-GTTGGCGTCTTTGATCACCT-3′ (downstream); p62/SQSTM1: 5′-CTGGGACTGAGAAGGCTCAC-3′ (upstream) and 5′-GCAGCTGATGGTTTGGAAAT-3′ (downstream); and RPL6: 5′-AGATTACGGAGCAGCG CAAGATTG-3′ (upstream) and 5′-GCAAACACAGATCGCAGGTAGCCC-3′ (downstream). *RPL6* mRNA was the reference on which all the other values were normalized because it remained substantially stable during all the treatments.

### 2.6. Immunoprecipitation

Immunoprecipitation was performed at RT for 2 hrs using 1 *μ*g of an anti-HuR antibody (Santa Cruz Biotechnology Inc.) per 50 *μ*g of cytoplasmic proteins diluted in the immunoprecipitation buffer (50 mM Tris pH 7.4, 150 mM NaCl, 1 mM MgCl_2_, 0.05% Igepal, 20 mM EDTA, 100 mM DTT, protease inhibitor cocktail, and RNAase inhibitor) in the presence of 50 *μ*l protein A/G plus agarose (Santa Cruz Biotechnology Inc.), according to a previously published protocol with minor modifications [15]. The sample, representing the immunoprecipitated HuR protein, was then subjected to either Western blotting with antibodies recognizing phosphorylated residues (anti-phospho-threonine or anti-phospho-serine, resp.) or RNA extraction. For each sample, 100 *μ*l of immunoprecipitation mix was taken and used as “input signals” to normalize the data in Western blotting or real-time qPCR. An irrelevant antibody (Santa Cruz Biotechnology Inc.) with the same isotype as the specific immunoprecipitating antibody served as a negative control.

### 2.7. Polysome RNA Extraction and Profile Analysis

ARPE-19 cells (3 × 10^6^ cells) were treated with either DMSO or MG132 + AICAR as described. Two hours after treatment, cells were incubated with 10 mg/ml cycloheximide (Sigma-Aldrich) for 5 min at 37°C and washed twice with cold PBS containing 1 mg/ml cycloheximide. Cells were scraped and lysed in fresh polysome buffer (10 mM NaCl, 10 mM MgCl_2_, 10 mM Tris–HCl pH 7.5, 1% Triton-X100, 1% Na-deoxycholate, 0.2 U/*μ*l RiboLock RNase inhibitor, 1 mM dithiothreitol, and 0.01 mg/ml cycloheximide). Cell lysates were centrifuged at 13000*g* for 10 min at 4°C. The supernatants were then layered onto 15/50% sucrose gradients (prepared in 300 mM Tris–HCl, 1 M NaCl, and 100 mM MgCl_2_) and centrifuged at 40000 rpm for 1.40 hrs at 4°C in SW41Ti Rotor. The gradients were fractionated using a Teledyne Isco gradient fractionator that continuously measured the absorbance at 260 nm. Fractions containing free RNA (pooled sample of fractions 3 and 4) subpolysomal, monosome (pooled sample of fractions 5 and 6), and polysomal RNA (pooled sample of fractions 7, 8, and 9) were prepared. Free RNA, monosome, and polysome samples were treated with proteinase K (100 *μ*g/ml) in 1% SDS for 1 hr at 37°C followed by extraction with 250 *μ*l volumes of phenol–chloroform and 1 mM NaCl and by precipitation in one volume of isopropanol for 30 min at 14000*g* and 4°C. The recovered RNA pellet was resuspended in 20 *μ*l of RNase-free water. Synthesis of cDNA was carried out on a RevertAid RT kit (Thermo Fisher Scientific, Waltham, MA, USA). Real-time qPCR analysis was performed with triplicates using 2XqPCR SybrGreen Mix Separate-Rox PB20 (PCR Biosystems, London, United Kingdom) on a CFX96-RT-PCR Detection system (Bio-Rad Laboratories, Watford, United Kingdom). Expression levels of *HuR* and *p62* were evaluated and normalized to free RNA. *GAPDH* was used as a housekeeping gene.

### 2.8. Immunocytochemistry

ARPE-19 cells (7500 cells/well) were seeded onto poly-L-lysin-coated plates for 48 hrs before exposures. Cells were pretreated or not with the compound C and then exposed to either AICAR, MG132, or both, for 2 hrs. Cells were fixed using 4% paraformaldehyde for 10 min, then incubated with a permeabilizing buffer (0.02% Triton-X100 in PBS) for 15 min, and incubated with 3% albumin for 45 min. Cells were incubated with the primary HuR antibody (at dilution 1 : 200) for 1 hr, and the secondary Alexa fluor 594 goat anti-mouse IgG (Life Technology, Thermo Fisher Scientific, Waltham, MA) (at dilution 1 : 500) for 1 hr; then cells were stained with DAPI (1 : 10,000) (Life Technology). The PerkinElmer image plate reader Operetta was used for imaging and the evaluation of HuR localization. The ratio between the cytoplasmic and nuclear signals of HuR was calculated as the mean of each ratio in every single cell in every well (triplicates). For higher magnification, immunofluorescence analysis was performed using a Zeiss Observer Z1 microscope equipped with Apotome module, with a Plan Apochromatic (63x, NA 1.4) objective. Images were acquired using Zen 1.1 (blue edition) imaging software (Zeiss, Milan, Italy) and assembled with ImageJ software.

### 2.9. ELISA Assay

Cells were quantitatively analyzed for phospho-SAPK/JNK using an ELISA kit (Cell Signaling) according to the manufacturer's instructions. The concentrations of phospho-SAPK/JNK were calculated from a standard curve and corrected for the protein concentration of each sample.

### 2.10. Statistical Analysis

Three independent experiments with 1–3 parallel samples were performed for each exposure. The statistical analyses were performed using the GraphPad InStat software. Results were analyzed by either the analysis of variance (ANOVA) or the nonparametric method followed by an appropriate post hoc test, as indicated in the figure legends. Differences were considered statistically significant when *p* < 0.05.

## 3. Results

### 3.1. AICAR and MG132 Cotreatment Leads to a Rapid HuR Protein Activation

We previously showed that in ARPE-19 cell line under 24 hr proteasomal inhibitor MG132, HuR protein binds *p62* mRNA; the specific involvement of HuR in p62 expression regulation at the posttranscriptional level was confirmed by the finding that the MG132-induced increase of p62 protein is counteracted in HuR-silenced ARPE-19 cells [18]. The addition of AICAR triggered autophagy by favoring the clearance of p62-conjugated protein aggregates, finally improving survival in 24 hr MG132-treated RPE cells [18]. In the present study, we aim to demonstrate that at early time points the AICAR + MG132 cotreatment activates HuR and upregulates p62 expression needed for the autophagy process.

**Figure 1:**
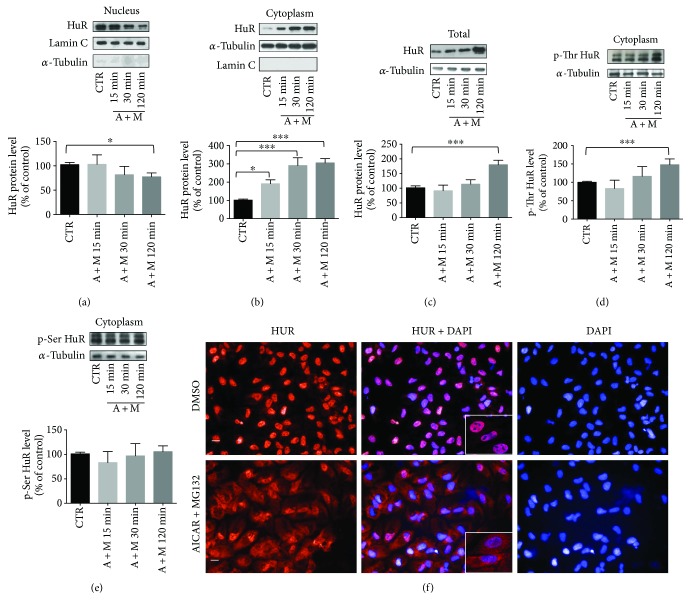
Translocation of HuR protein following AICAR + MG132 exposure. (a) Representative Western blotting (upper) and densitometric analysis (lower) of HuR protein levels in the nucleus (a), cytoplasm (b), and total homogenate (c) of ARPE-19 cells exposed to either solvent (CTR) or AICAR + MG132 (A + M) for increasing times (15, 30, and 120 min). Optical densities of HuR bands were normalized to lamin C for the nucleus and to *α*-tubulin for both the cytoplasm and total homogenate. The same proteins were also used as purity controls for each cellular fraction. The values are expressed as mean percentages + S.E.M. (*n* = 3–6; ^∗^ *p* < 0.05 and ^∗∗∗^ *p* < 0.0001; Dunnett's multiple comparison test). (d, e) Representative Western blotting (upper) and densitometric analyses (lower) of HuR protein phosphorylated in threonine residues (p-Thr HuR; (d)) and serine residues (p-Ser HuR; (e)) in the cytoplasm of ARPE-19 cells exposed to either solvent (CTR) or AICAR + MG132 (A + M) for increasing times (15, 30, and 120 min). Optical densities of phosphorylated HuR bands were normalized to *α*-tubulin (loading control detected in the input signals), and the results expressed as mean percentages + S.E.M. (*n* = 3–6; ^∗∗∗^ *p* < 0.0001; Dunnett's multiple comparison test). (f) Representative immunocytochemistry images of HuR protein in ARPE-19 cells exposed to either solvent (CTR) or AICAR + MG132 for 2 hrs. The left panels show HuR staining (red), the right panels show nuclei staining with DAPI (blue), and the middle panels merged images. Imaging (40x magnification) was made with a PerkinElmer image plate reader Operetta. Scale bar: 20 *μ*m. Inserts: immunofluorescence analysis of HuR was performed using a Zeiss Observer Z1 microscope equipped with Apotome module, with a Plan Apochromatic (63x, NA 1.4) objective. Nuclei staining with DAPI (blue). Images were acquired using Zen 1.1 (blue edition) imaging software and assembled with ImageJ software.

ARPE-19 cells were exposed concurrently to AICAR and MG132 (A + M) for increasing times (15 min, 30 min, and 2 hrs), to study the early events of the HuR-p62 pathway under proautophagy conditions. Since both the abundance and subcellular localization of HuR protein are key determinants for its activity, we first evaluated the HuR levels in both nuclear and cytoplasmic fractions after A + M. We found that the cotreatment triggered a rapid HuR translocation from the nucleus to the cytoplasm, evident after 15 min and statistically significant in both the cellular fractions after 2 hr exposure (Figures 1(a) and 1(b)). Immunocytochemistry experiments confirmed that HuR content was elevated in the cytoplasm of ARPE-19 cells after 2 hr A + M (Figure 1(f)). At this time, a significant increase of total HuR protein level (Figure 1(c)), associated with a higher phosphorylation of HuR in threonine residues in the cytoplasm (Figure 1(d)), was also found. Conversely, at all the times considered, no significant changes in phosphorylated serine residues of HuR were detected (Figure 1(e)). For this, in the following experiments, we focused on HuR threonine phosphorylation.

**Figure 2:**
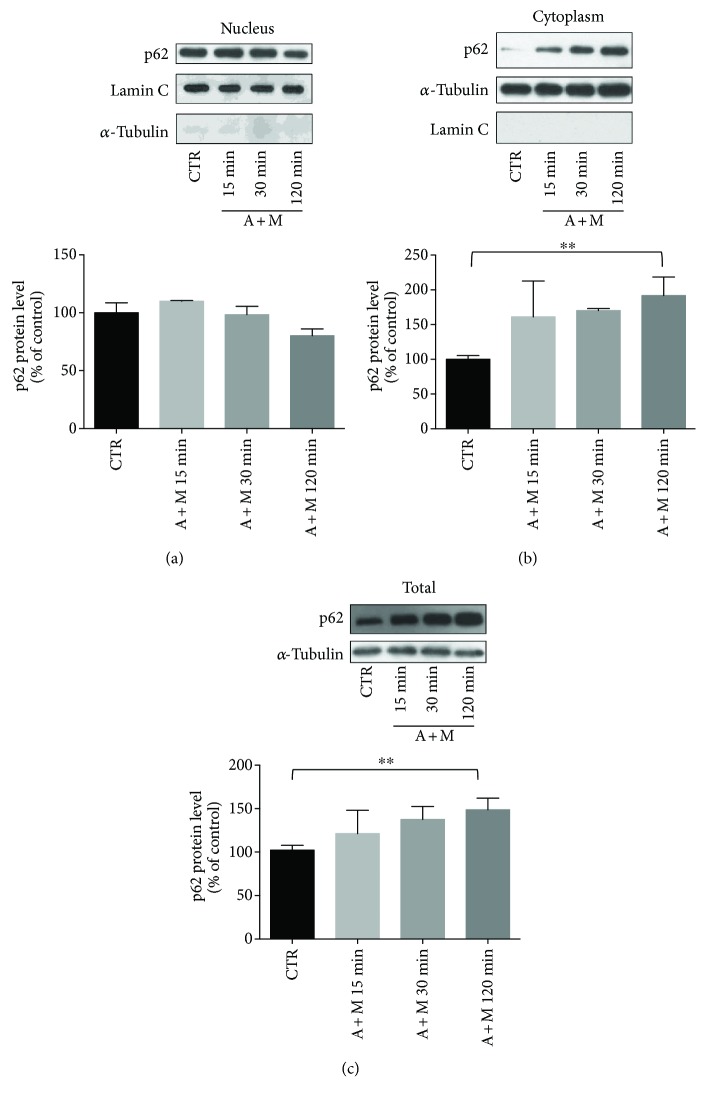
Evaluation of p62 protein level following AICAR + MG132 exposure. (a) Representative Western blotting (upper) and densitometric analysis (lower) of p62 protein levels in the nucleus (a), cytoplasm (b), and total homogenate (c) of ARPE-19 cells exposed to either solvent (CTR) or AICAR + MG132 (A + M) for increasing times (15, 30, and 120 min). Optical densities of p62 bands were normalized to lamin C for the nucleus and to *α*-tubulin for both the cytoplasm and total homogenate. The same proteins were also used as purity controls for each cellular fraction. The values are expressed as mean percentages + S.E.M. (^∗∗^ *p* < 0.005, *n* = 4; Dunnett's multiple comparison test).

We then evaluated possible changes in p62 protein levels in the nucleus, cytoplasm, and total lysate at all the times considered (15 min, 30 min, and 2 hrs) (Figures 2(a)–2(c)). We found that 2 hr A + M-treated ARPE-19 cells showed a significant increase of p62 in the cytoplasm (Figure 2(b)), together with a higher p62 total content than control cells (Figure 2(c)). In contrast, the 2 hr treatment with either MG132 or AICAR alone was not able to increase the p62 protein levels [mean ± S.E.M.; CTR: 808.4 ± 48.7; MG132: 1078.0 ± 143.3; AICAR: 894.7 ± 123.4; not significant (N.S.); A + M: 1496.0 ± 208.5; *p* < 0.05 for A + M versus CTR; *n* = 7, Dunn's multiple comparisons test]. According to these results, the 2 hr A + M cotreatment was selected for all the following experiments.

In addition, to confirm that A + M triggers autophagy, we measured the early marker of autophagy beclin-1, finding a slight but significant increase of its protein level (Supplementary Figure 1, A), coherent with the first steps of autophagy that require the synthesis of effectors to be properly induced [29]. Even the 24 hr A + M cotreatment did not affect the viability of the ARPE-19 cell, as demonstrated by the LDH assay (Supplementary Figure 1, B).

### 3.2. HuR Binds to *p62* Transcript and Positively Affects Its New Protein Synthesis under AICAR and MG132 Cotreatment

**Figure 3:**
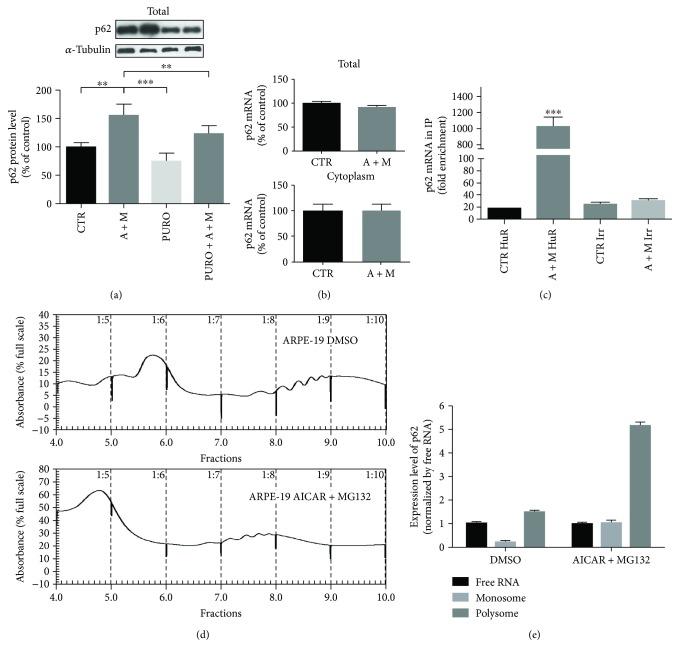
Levels of *p62* mRNA, its binding by HuR protein, and de novo translation following AICAR + MG132 exposure. (a) Representative Western blotting (upper) and densitometric analyses (lower) of p62 protein levels in the total homogenates of ARPE-19 cells exposed to either solvent (CTR) or AICAR + MG132 (A + M) for 2 hrs in the presence or not of puromycin (1 *μ*M, PURO). Optical densities of p62 bands were normalized to *α*-tubulin, and the results expressed as mean percentages + S.E.M. (*n* = 6; ^∗∗^ *p* < 0.001 and ^∗∗∗^ *p* < 0.0001; Tukey's multiple comparisons test). (b) Determination by real-time qPCR of *p62* mRNA levels in the total homogenate (upper) and cytoplasm (lower) of ARPE-19 cells exposed to either solvent (CTR) or AICAR + MG132 (A + M) for 2 hrs. *p62* mRNA levels were normalized in accordance with the corresponding *RPL6* mRNA content. The values are expressed as mean percentages + S.E.M. The experiments were performed in duplicate on 4-5 independent sets of cells (^∗∗^ *p* < 0.01, Student's *t* test). (c) Fold enrichment detected by real-time qPCR of *p62* mRNA following immunoprecipitation (IP) with either anti-HuR antibody or irrelevant antibody (Irr) in the cytoplasm of ARPE-19 cells exposed to either solvent (CTR) or AICAR + MG132 (A + M) for 2 hrs (*n* = 3; ^∗∗∗^ *p* < 0.0001; Tukey's multiple comparison test). (d) Polysome profile of *p62* mRNA was determined using 15–50% sucrose gradient sedimentation. (e) Real-time qPCR analysis and transcript level quantification for *p62* were performed in free RNA (pooled fractions 3 and 4), monosomes (pooled fractions 5 and 6), and polysome (pooled fractions 7, 8, and 9) of ARPE-19 cells treated with either solvent (DMSO) or AICAR + MG132 for 2 hrs. Relative expression of *p62* was normalized to mRNA of free RNA sample, considering the value of *GAPDH* as a housekeeping gene.

To evaluate whether the increased cytoplasmic p62 level following the 2 hr A + M cotreatment was due to de novo protein synthesis, we measured by Western blotting p62 protein levels in total homogenates of ARPE-19 cells exposed or not to puromycin, an inhibitor of protein synthesis. We found that the A + M cotreatment led to a significant p62 protein upregulation that was prevented by puromycin (Figure 3(a)), indicating that new p62 protein synthesis occurred in this condition. Interestingly, following A + M, total homogenates of ARPE-19 cells displayed also augmented HuR protein level, an effect that was blocked by puromycin (Supplementary Figure 2, A). To investigate whether A + M also favored the *p62* transcription, we measured by real-time qPCR *p62* mRNA content in both total homogenate and the cytoplasmic fraction of ARPE-19 cells, finding no changes in the *p62* mRNA expression (Figure 3(b)). As well, total *HuR* mRNA content was not affected by the cotreatment (Supplementary Figure 2, B). Notably, A + M promoted HuR protein binding to *p62* mRNA in the cytoplasmic fraction of ARPE-19 cells (Figure 3(c)). In basal conditions, the physical association between HuR protein and *p62* mRNA was almost absent, being the content of *p62* transcript in the immunoprecipitated HuR of control cells as low as the one observed for an immunoprecipitating irrelevant antibody (Figure 3(c)). Finally, polysome profiling of *p62* mRNA during the A + M cotreatment showed a massive shift of this transcript on heavy polysomes from monosomes or free RNA fractions (Figures 3(d) and 3(e)). We found the same for *HuR* transcript (Supplementary Figure 3). Together, these data indicate that during the A + M treatment at 2 hours, both p62 and HuR proteins increase their expression levels by an exquisitely posttranscriptional mechanism inducing their de novo translation.

### 3.3. AICAR and MG132 Cotreatment Activates Erk1/2 Mediating HuR Cytoplasmic Increase, HuR Phosphorylation, and p62 Protein Upregulation

It is known that phosphorylation of HuR protein can affect its cellular localization and/or activity [30]. To study in further detail the effects of the A + M cotreatment on HuR activation, we evaluated the nucleocytoplasmic shuttling of HuR protein and its phosphorylation status in the presence of some kinase inhibitors.

**Figure 4:**
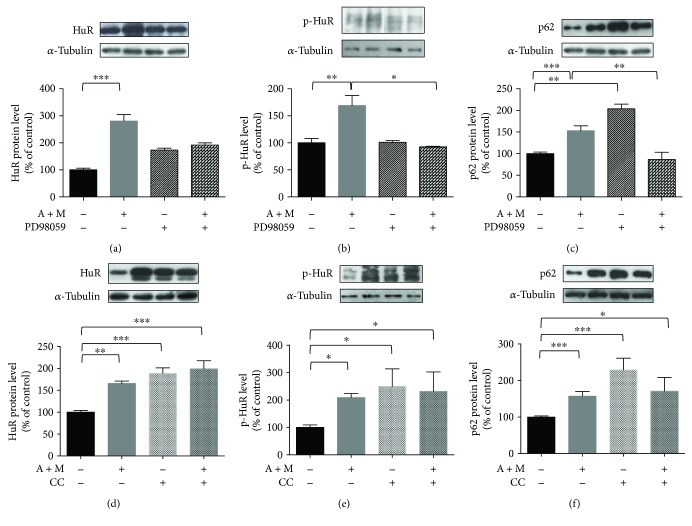
Effects of Erk1/2 and AMPK inhibitors on HuR translocation, its phosphorylation, and p62 expression. Representative Western blotting (upper) and densitometric analysis (lower) of levels of HuR (a, d), phospho-HuR (p-HuR, in threonine residues) (b, e), and p62 (c, f), in the cytoplasm of ARPE-19 cells exposed to either solvent or AICAR + MG132 (A + M) for 2 hrs, in the presence or not of Erk1/2 inhibitor (50 *μ*M PD98059) (a–c) or AMPK inhibitor (5 *μ*M compound C (CC)) (d–f). Optical densities of HuR, phospho-HuR (p-HuR, in threonine residues), and p62 bands were normalized to *α*-tubulin, and the results expressed as mean percentages + S.E.M. (*n* = 3–6; ^∗^ *p* < 0.05, ^∗∗^ *p* < 0.001, and ^∗∗∗^ *p* < 0.0001; Tukey's multiple comparison test).

Literature data on different cellular models reported that MG132 determines the Erk1/2 activation [31] and that Erk1/2 regulates the cytoplasmic translocation of HuR [32]. Thus, we first investigated the possible activation of Erk1/2 in our experimental conditions. We found a significant increase of phosphorylated Erk1/2 (p-Erk1/2) following the 2 hr A + M cotreatment in comparison to control cells (Supplementary Figure 4, A); interestingly, already at 30 min, A + M led to significantly higher p-Erk1/2 levels when compared to control (Supplementary Figure 4, B). The increase of p-Erk1/2 at 2 hrs was abolished by PD98059 (MEK-Erk inhibitor), which led downstream to a complete absence of a detectable p-Erk1/2 signal in all samples (Supplementary Figure 4, A). The cytoplasmic accumulation of HuR following the A + M exposure was compromised when the MEK/Erk1/2 pathway was inhibited (Figure 4(a)), being PD98059 responsible for HuR staying inside the nucleus (Supplementary Figure 4, C). This suggests the importance of Erk1/2 activation in the HuR translocation. The increase in phosphorylated HuR (p-HuR, in threonine residues) levels observed following A + M was not detectable when PD98059 was added (Figure 4(b)). Interestingly, PD98059 also impeded the p62 upregulation occurring under the A + M cotreatment (Figure 4(c)). ARPE-19 cells exposed to PD98059 alone showed a cytoplasmic/nuclear distribution of HuR protein mostly comparable to control (Supplementary Figure 4, C), while increased p62 protein levels were observed in the cytoplasm (Figure 4(c)).

### 3.4. Cytoplasmic HuR Increase and p62 Protein Upregulation Are Favored by AMPK Inhibition

Considering the well-known role of AMPK as a positive regulator of autophagy and the fact that AICAR is an AMPK activator, we evaluated on HuR and p62 the effects of the A + M cotreatment with/without AMPK inhibition. It was previously reported that AMPK favors HuR nuclear import by phosphorylating the HuR-mediating transport protein [33]. Accordingly, our Western blotting analyses showed that blocking AMPK by the compound C (CC) further potentiated the cytoplasmic accumulation of HuR triggered by A + M (Figure 4(d)), possibly explaining the trend to the increase of p-HuR observed in this compartment (Figure 4(e)). Immunocytochemistry experiments (Supplementary Figure 5) confirmed that in the presence of CC alone HuR content was unchanged in the nucleus and increased in the cytoplasm, respectively. Interestingly, the AMPK activator AICAR, alone or together with CC ± MG132, determined the HuR nuclear export and cytoplasmic accumulation. Therefore, AICAR's effect on the HuR nuclear-cytoplasmic shuttling is independent of AMPK activation. The blockade of AMPK resulted in increased cytoplasmic p62 protein level, which was even more pronounced when CC was given alone with respect to A + M (Figure 4(f)).

**Figure 5:**
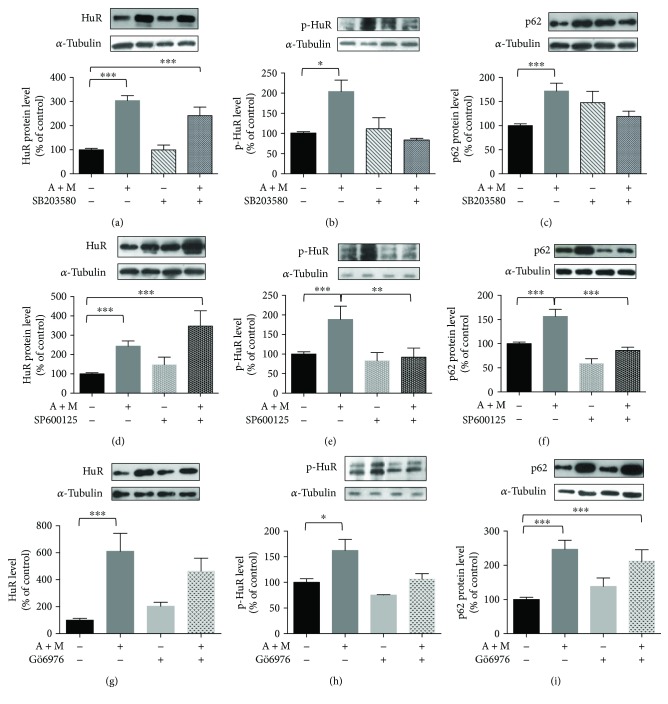
Effects of p38^MAPK^, JNK, and cPKC inhibitors on HuR translocation, its phosphorylation, and p62 expression. Representative Western blotting (upper) and densitometric analysis (lower) of levels of HuR (a, d, g), phospho-HuR (p-HuR) (b, e, h), and p62 (c, f, i), in the cytoplasm of ARPE-19 cells exposed to either solvent or AICAR + MG132 (A + M) for 2 hrs, in the presence or not of p38^MAPK^ inhibitor (50 *μ*M SB203580) (a–c), JNK inhibitor (10 *μ*M SP600125) (d–f), or cPKC inhibitor (2 *μ*M Gö6976) (g–i). Optical densities of HuR, phospho-HuR (p-HuR, in threonine residues), and p62 bands were normalized to *α*-tubulin, and the results expressed as mean percentages + S.E.M. (*n* = 3–6; ^∗^ *p* < 0.05, ^∗∗^ *p* < 0.001, and ^∗∗∗^ *p* < 0.0001; Tukey's multiple comparison test).

Some evidence from the literature shows that both the nuclear export and the phosphorylation of HuR are regulated by p38^MAPK^, a kinase important for the cell stress response [34, 35]. The inhibition of p38^MAPK^ by SB203580 did not affect the HuR export to the cytoplasm induced by A + M (Figure 5(a)), suggesting that, in our conditions, p38^MAPK^ was not primarily involved in the HuR nucleocytoplasm shuttling. However, SB203580 decreased the p-HuR levels in the cytoplasm (Figure 5(b)) and also counteracted the increased levels of p62 under the A + M cotreatment, although without statistical significance (Figure 5(c); A + M + SB203580 versus A + M *p* = 0.07).

### 3.5. Inhibition of JNK, but Not PKC, Counteracts p62 Increase under the AICAR and MG132 Cotreatment

JNK plays an important role in cellular response to a variety of stimuli. Previous studies found that JNK activation regulates the p62 expression in different contexts [31, 36, 37]. Therefore, the role of JNK in the modulation of p62 protein expression under the A + M cotreatment was examined. First, by ELISA, we found that cytoplasmic JNK is activated upon our proautophagy stimulus (Supplementary Figure 6). The phosphorylation of HuR, but not its accumulation in the cytoplasm, was affected by the JNK inhibitor SP600125 (Figures 5(d) and 5(e)), suggesting that JNK may be a new kinase regulating HuR. SP600125 alone resulted in significantly decreased p62 protein levels in the cytoplasm with respect to control (*p* < 0.05); SP600125 also prevented the increase of p62 upon the A + M cotreatment (Figure 5(f)).

Considering that HuR protein is a target of conventional PKC isoforms (cPKC) [15, 38] and that PKC is often an upstream regulator of other kinases, including Erk1/2 [27], we investigated the effects of cPKC blocking by Gö6976. Basal p-Erk1/2 was inhibited by Gö6976; in contrast, Erk1/2 activation under the A + M cotreatment seems to be PKC-independent since p-Erk1/2 remained unchanged upon the concomitant administration of Gö6976 to ARPE-19 cells (Supplementary Figure 7). As expected, the cytoplasmic increase of HuR protein was not affected by the PKC inhibition in A + M-treated ARPE-19 cells, although we observed decreased levels of p-HuR (Figures 5(g) and 5(h)). No significant alteration in the p62 protein content was observed in A + M-treated cells also exposed to Gö6976 (Figure 5(i)). These data suggest that PKC is not necessary for the A + M-induced p62 upregulation.

## 4. Discussion and Conclusion

Autophagy is a highly coordinated process that is regulated at several levels, including protein-protein interactions and transcriptional control, both representing the main concerns of most studies on the regulation of autophagy. Conversely, although it is becoming clear that a “whole-cell view” of autophagy is needed to understand better the molecular basis of its regulation [39], posttranscriptional mechanisms controlling the gene expression in autophagy are mainly unknown. In this study with ARPE-19 cells, we provide information on the early effects of a proautophagy stimulus on the RNA-binding HuR protein and p62, whose mRNA we previously demonstrated to be a HuR's target [18].

As known, p62 acts as a carrier for protein degradation in the autophagy machinery and its levels change in the function of the stimuli and the autophagy phases; p62 levels increase when autophagy needs to be triggered, and they decrease when autophagy is fully activated since p62 itself is degraded by autophagy [40, 41]. In our previous publication [18], we showed that a long exposure (24 hours) of ARPE-19 cells to the AICAR + MG132 cotreatment activates autophagy flux, leading to a consequent decrease of p62 protein content, clearance of protein aggregates, and improvement in cell viability. In the present research, we aimed to dissect the early phases following the AICAR + MG132 cotreatment, in particular, the activation of HuR and the upregulation of p62 that is required for triggering the autophagy process.

First, we here demonstrate that the proautophagy A + M cotreatment promotes HuR protein translocation from the nucleus to the cytoplasm, which is observable already after a few minutes (15 and 30 min), and reaches statistical significance at 2 hrs. In parallel, a dramatic increase of the binding between HuR protein and *p62* mRNA in the cytoplasm is observed after the 2 hr A + M exposure, being almost absent in basal conditions. No change in the *p62* mRNA level is observed, indicating that *p62* transcription does not occur. We thought that the binding of HuR protein to *p62* mRNA could affect its translation, not its stability, as previously reported for another HuR's target transcript (*VEGF*) in human HeLa cell line [42]. Consistently, after 2 hr A + M, we found a significant increase of *p62* and *HuR* mRNAs on heavy polysomes and that both protein levels increase in the cytoplasm and whole lysate of ARPE-19 cells; this effect is prevented by inhibiting protein synthesis, further supporting that de novo p62 protein translation occurs. These effects at the polysomal level may be mediated by HuR, although future studies on HuR-*p62* mRNA association in HuR-deficient cells will be needed to confirm our hypothesis. In agreement with our results on p62, a recent study demonstrated that H_2_O_2_ exposure enhances the autophagic pathway together with increased p62 protein levels in RPE cells as an early prosurvival response against oxidative stress [43]. As expected in the timeframe here being considered, besides increased levels of p62 protein, after 2 hr A + M, we also found a slight upregulation of beclin-1, an early marker of autophagy activation.

**Figure 6:**
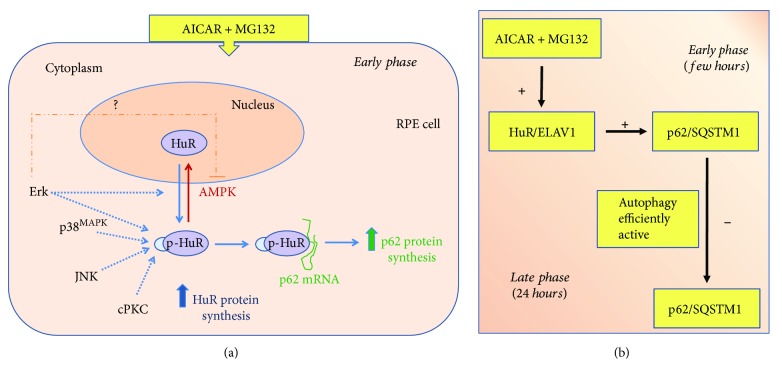
Hypothesis of the flowchart induced by AICAR + MG132 in RPE cells. (a) According to our findings, AICAR + MG132 cotreatment induces an early translocation of HuR protein from the nucleus to the cytoplasm, accompanied by an increase of its phosphorylation in threonine residues. The activated HuR protein binds to *p62* mRNA and favors its translation, upregulating p62 protein. As well, HuR protein expression is increased in this condition. Both AICAR + MG132-mediated HuR shuttling and phosphorylation are prevented by Erk inhibitor, and this possibly reverberates on p62 levels. Vice versa, AMPK is involved in HuR nucleus import, and AMPK inhibition favors both HuR permanence in the cytoplasm and p62 increase. The AICAR + MG132-induced phosphorylation of HuR is affected by inhibitors of p38^MAPK^, JNK, and cPKC. p38^MAPK^ and JNK inhibitors seem to contrast p62 increase under AICAR + MG132 cotreatment, while PKC inhibitor has no substantial effect. For further details, see the text. (b) The ideal temporal sequela of the events with the difference between the early and late effects induced by the AICAR + MG132 cotreatment, based on present results and our previous publication [18].

The A + M-mediated nucleocytoplasmic shuttling of HuR is also accompanied by the new synthesis of HuR protein at 2 hrs, in line with the previous *in vitro* observation in human SH-SY5Y cells for the ELAV member upregulation [44]. After 2 hr A + M, an increase in cytoplasmic HuR phosphorylation status, specifically in threonine residues, also occurs. With the aim to identify the pathways potentially mediating these effects on HuR and finally affecting p62 expression, we studied the involvement of various kinases (Erk1/2, AMPK, p38^MAPK^, JNK, and PKC). The main findings and final hypotheses are reported in Table 1 and Figure 6, respectively.

Distinct subfamilies of MAPK include Erk1/2, p38^MAPK^, and JNK, which can be activated in response to diverse extracellular stimuli [45]. We found that A + M induces the activation of Erk1/2, which contributes to HuR nuclear export and cytoplasmic accumulation and to a parallel increase in p62 protein level; both A + M-mediated effects are prevented by PD98059. These findings are in agreement with the literature reporting that Erk1/2 regulates the HuR nucleocytoplasmic shuttling in hepatic cells [32] and it increases the p62 expression in various cell types [30]. Likewise, in hepatocytes, AICAR treatment favors the HuR binding to its target mRNA in an Erk1/2-dependent manner [46]. In our context, cytoplasmic p-HuR levels following the A + M treatment are also decreased by PD98059, suggesting that Erk1/2 is important for HuR/p62 pathway activation in ARPE-19 cells.

It was previously reported that AMPK indirectly regulates the HuR nucleocytoplasmic shuttling, promoting HuR nuclear import in intestinal epithelial cells [33]. In agreement with this, we found that inhibiting AMPK by CC (in both presence and absence of the A + M stimulus) favors HuR cytoplasmic accumulation and, therefore, p62 increase.

Erk1/2 inhibits AMPK in a tissue- and context-dependent manner [47], so we may also hypothesize that A + M triggers Erk1/2 activation, which in turn inhibits AMPK, finally leading to HuR cytoplasmic accumulation and p62 increase.

PKC is upstream of Erk1/2, and PKC activation induces the activation of the Raf/MEK/Erk1/2 pathway [27, 48, 49]. However, we have to point out that the axis PKC-Erk1/2 is not widely spread and, where it is present, functional outcomes of the PKC-induced Raf-MEK-Erk1/2 cascade activation are both cell type-specific and PKC isoform-specific [50, 51]. We found that in ARPE-19 cells, Erk1/2 activation is PKC-dependent in basal condition, but PKC-independent under the A + M exposure, since no change in p-Erk1/2 is observed when the proautophagy stimulus is coadministered with Gö6976. These findings may be explained by considering that AICAR can directly activate Erk1/2 [46], thus possibly bypassing the PKC inhibition. In our condition, Gö6976 affects the HuR phosphorylation but not its shuttling, which is in agreement with a previous observation on ELAV proteins [44]. Given that the PKC-*γ* isoform is not expressed in RPE cells [52], we hypothesize the involvement of PKC-*α*, or PKC-*β*I/II in HuR phosphorylation and/or Erk1/2 modulation. However, the increase in p62 levels under the A + M coexposure seems to be PKC-independent since it also occurs in the presence of Gö6976.

Previous studies showed that p38^MAPK^ signaling is involved in the p62 expression via Nrf2 transcription and that the pharmacological inhibition of p38^MAPK^ reduces p62 levels in fibroblasts exposed to oxidative stress [53]. In other cellular models, it was reported that the p38^MAPK^ activation leads to HuR phosphorylation, cytoplasmic accumulation, and enhanced binding to its target mRNAs [34, 35]. We observed that, when SB203580 is added to A + M, impaired phosphorylation of HuR, but not HuR cytoplasmic increase, is observed. This may reveal that p38^MAPK^ is more involved in the HuR phosphorylation than in its nucleocytoplasm shuttling. We also found that blocking p38^MAPK^ prevents the p62 increase, by possibly acting on its posttranscriptional control via HuR.

Finally, we evaluated the involvement of another MAPK, JNK, that has been suggested to regulate p62 expression via Nrf2 in human hepatoma cells [54]. We found that JNK is activated under A + M and that SP600125 causes suppressed levels of both p62 and p-HuR, suggesting that JNK may promote the accumulation of p62 also at posttranscriptional level through HuR. Interestingly, to our knowledge, there is no evidence in the literature on a possible link between JNK and HuR. Our findings represent the first clue on this topic.

Based on literature and our present findings, we hypothesize the involvement of Erk1/2, p38^MAPK^, and JNK kinases in HuR activation and p62 expression, and we suggest that alterations in these pathways may be relevant for AMD. Future studies evaluating in more detail the effects of these kinase modulators on HuR-p62 binding and p62 translational efficiency will be of interest to confirm the relevance of these cascades in RPE.

A growing body of literature indicates that autophagy impairment plays a role in the AMD pathogenesis and that the modulation of autophagy and related signaling pathways may provide novel therapeutic strategies for human disease prevention or treatment, including ocular diseases [23, 55–61]. Due to the complexity of mechanisms regulating this process, the modulation of autophagy is a challenging field of research. Interestingly, no molecules directly targeting the autophagy machinery are currently in clinical trials; the majority of the compounds under such studies indeed affect the regulation of autophagy [61]. Autophagy-regulating kinases have been proposed as potential therapeutic targets for AMD [62]. For instance, a key role for Erk1/2, as well as AMPK, in AMD has been suggested [63]. Moreover, recent studies have confirmed the importance of HuR in various ocular pathologies [64, 65] and laid the foundation for the druggability assessment of HuR protein [66]; compounds able to directly act on the HuR protein and *p62* mRNA complex formation may thus represent new potential tools regulating p62 content.

In conclusion, our study supports the importance of the HuR-p62 pathway and the autophagy-regulating kinases as potential therapeutic targets for AMD.

## Figures and Tables

**Table 1:** Effects of specific kinase inhibitors on HuR cytoplasmic accumulation, its threonine phosphorylation, and p62 increase, compared to the AICAR + MG132 cotreatment.

	Kinase targeted by inhibition	HuR cytoplasmic accumulation	HuR phosphorylation	p62 protein levels
AICAR + MG132		↑	↑	↑
	**Erk1/2**	↓	↓	↓
	**AMPK**	—	—	—
	**p38** ^**MAPK**^	—	↓	↓
	**JNK**	—	↓	↓
	**cPKC**	—	↓	—

↑: increase; ↓: decrease; —: no variation. For further details, see the text.

## Supplementary Materials

Supplementary 1 Effects on beclin-1 expression and cell viability following AICAR + MG132 exposure. (A) Representative Western blotting (upper) and densitometric analysis (lower) of beclin-1 protein levels in the cytoplasm of ARPE-19 cells exposed to either solvent (CTR) or AICAR and MG132 (A + M) for 2 hrs. Optical densities of beclin-1 bands were normalized to *α*-tubulin, and the results expressed as mean percentages + S.E.M. (*n* = 6; ^∗∗^ *p* < 0.001; *n* = 7; Student's *t*-test). (B) Absorbance values relative to cell viability measured by LDH assay in ARPE-19 cells exposed to either solvent (CTR) or AICAR and MG132 (A + M) for 24 hrs. Results expressed as mean percentages + S.E.M. versus control (100%) (*n* = 6; N.S.).

Supplementary 2 Evaluation of HuR protein and mRNA levels following AICAR + MG132 exposure. (A) Representative Western blotting (upper) and densitometric analyses (lower) of HuR protein levels in the total homogenates of ARPE-19 cells exposed to either solvent (CTR) or AICAR + MG132 (A + M) for 2 hrs in the presence or not of puromycin (1 *μ*M, PURO). Optical densities of HuR bands were normalized to *α*-tubulin, and the results expressed as mean percentages + S.E.M. (*n* = 3; ^∗^ *p* < 0.05; ^∗∗∗^ *p* < 0.0001; Tukey's multiple comparison test). (B) Determination by real-time qPCR of *HuR* mRNA levels in the total homogenate of ARPE-19 cells exposed to either solvent (CTR) or AICAR + MG132 (A + M) for 2 hrs. *HuR* mRNA levels were normalized in accordance with the corresponding *RPL6* mRNA content. The values are expressed as mean percentages + S.E.M. The experiments were performed in duplicate on 4-5 independent sets of cells.

Supplementary 3 AICAR + MG132 induces de novo translation of *HuR* mRNA. ARPE-19 cells were treated with either solvent (DMSO) or AICAR + MG132 for 2 hrs followed by polysome separation and Real-time qPCR analysis. *HuR* transcript level quantification was performed in free RNA, monosomes, and polysome of ARPE-19 cells. Relative expression of *HuR* was normalized to mRNA of free RNA sample, considering the value of *GAPDH* as a housekeeping gene.

Supplementary 4 Effects on Erk1/2 activation following AICAR + MG132 exposure in the presence of Erk1/2 inhibitor. (A) Representative Western blotting (upper) and densitometric analyses (lower) of phospho-Erk1/2 (p-Erk) protein levels in the cytoplasm of ARPE-19 cells exposed to either solvent or AICAR + MG132 (A + M), in the presence or not of Erk1/2 inhibitor (50 *μ*M PD98059) for 2 hrs. Optical densities of p-Erk bands were normalized to *α*-tubulin, and the results are expressed as mean percentages + S.E.M. (*n* = 4; ^∗∗∗^ *p* < 0.0001; Tukey's multiple comparison test). (B) Representative Western blotting (upper) and densitometric analyses (lower) of phospho-Erk1/2 (p-Erk) protein levels in the cytoplasm of ARPE-19 cells exposed to either solvent or AICAR + MG132 (A + M) for 30 min. Optical densities of p-Erk bands were normalized to *α*-tubulin, and the results are expressed as mean percentages + S.E.M. (*n* = 4; ^∗∗∗^ *p* < 0.0001; Student's *t*-tests). (C) The mean ratio + S.E.M. between the cytoplasmic and nuclear signals of HuR protein in the cytoplasm of ARPE-19 cells exposed to either solvent or AICAR + MG132 (A + M), in the presence or not of Erk1/2 inhibitor (50 *μ*M PD98059) for 2 hrs (*n* = 3; ^∗^ *p* < 0.05; Dunnett's multiple comparisons test).

Supplementary 5 Effects of AMPK inhibitor on HuR translocation. (A) Immunocytochemistry of ARPE-19 cells exposed to either solvent (CTR) or AICAR + MG132 alone or together, in the presence or not of AMPK inhibitor (5 *μ*M CC) for 2 hrs. The left panels of immunocytochemistry show HuR staining (red), the right panels show nuclei staining with DAPI (blue), and the middle panels show merged images. Scale bar: 20 *μ*m. Inserts: immunofluorescence analysis of HuR was performed using a Zeiss Observer Z1 microscope equipped with Apotome module, with a Plan Apochromatic (63x, NA 1.4) objective. Nuclei staining with DAPI (blue). Images were acquired using Zen 1.1 (blue edition) imaging software and assembled with ImageJ software. (B) The ratio between the cytoplasmic and nuclear signals of HuR is calculated as the mean of each ratio in each single cell in every well (*n* = 3; ^∗^ *p* < 0.05 and ^∗∗^ *p* < 0.005; Dunnett's multiple comparison test). Imaging (40x magnification) and evaluation of HuR localization were made with the PerkinElmer image plate reader Operetta.

Supplementary 6 Effects on JNK activation following AICAR + MG132 exposure. Levels of phosphorylated JNK in the cytoplasm of ARPE-19 cells exposed for 2 hrs to either solvent (CTR) or AICAR (A + M) measured by ELISA (*n* = 4; ^∗∗∗^ *p* < 0.0001; Student's *t*-test).

Supplementary 7 Effects of cPKC inhibitor on Erk activation. Representative Western blotting (upper) and densitometric analyses (lower) of phospho-Erk1/2 (p-Erk) protein levels in the cytoplasm of ARPE-19 cells exposed to either solvent or AICAR (A + M), in the presence or not of cPKC inhibitor (2 *μ*M Gö6976) for 2 hrs. Optical densities of p-Erk bands were normalized to *α*-tubulin, and the results are expressed as mean percentages + S.E.M. (^∗∗^ *p* < 0.005; *n* = 4; Tukey's multiple comparison test).
