# Supplementary figures and images for: Autophagy Stimulus Promotes Early HuR Protein Activation and p62/SQSTM1 Protein Synthesis in ARPE-19 Cells by Triggering Erk1/2, p38MAPK, and JNK Kinase Pathways
Source: Oxid Med Cell Longev. 2018 Feb 8;2018:4956080. doi: 10.1155/2018/4956080 (PMC5822911; doi:10.1155/2018/4956080)

## Slide 1
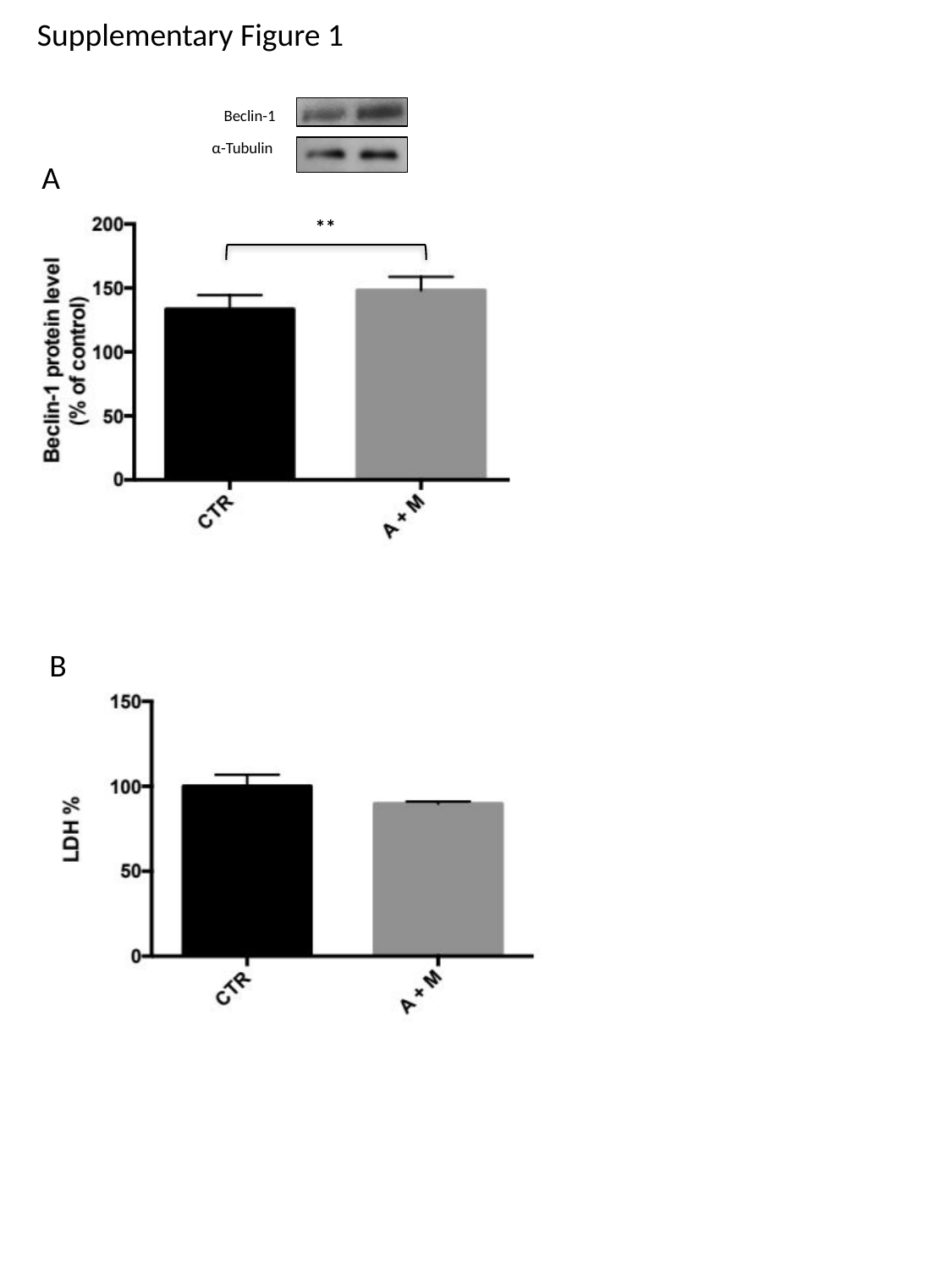

Supplementary Figure 1
Beclin-1
α-Tubulin
A
**
B

Supplement: Supplementary 1 — Effects on beclin-1 expression and cell viability following AICAR + MG132 exposure. (A) Representative Western blotting (upper) and densitometric analysis (lower) of beclin-1 protein levels in the cytoplasm of ARPE-19 cells exposed to either solvent (CTR) or AICAR and MG132 (A + M) for 2 hrs. Optical densities of beclin-1 bands were normalized to α-tubulin, and the results expressed as mean percentages + S.E.M. (n = 6; ∗∗ p < 0.001; n = 7; Student's t-test). (B) Absorbance values relative to cell viability measured by LDH assay in ARPE-19 cells exposed to either solvent (CTR) or AICAR and MG132 (A + M) for 24 hrs. Results expressed as mean percentages + S.E.M. versus control (100%) (n = 6; N.S.). [file 4956080.f1.pptx]

## Slide 1
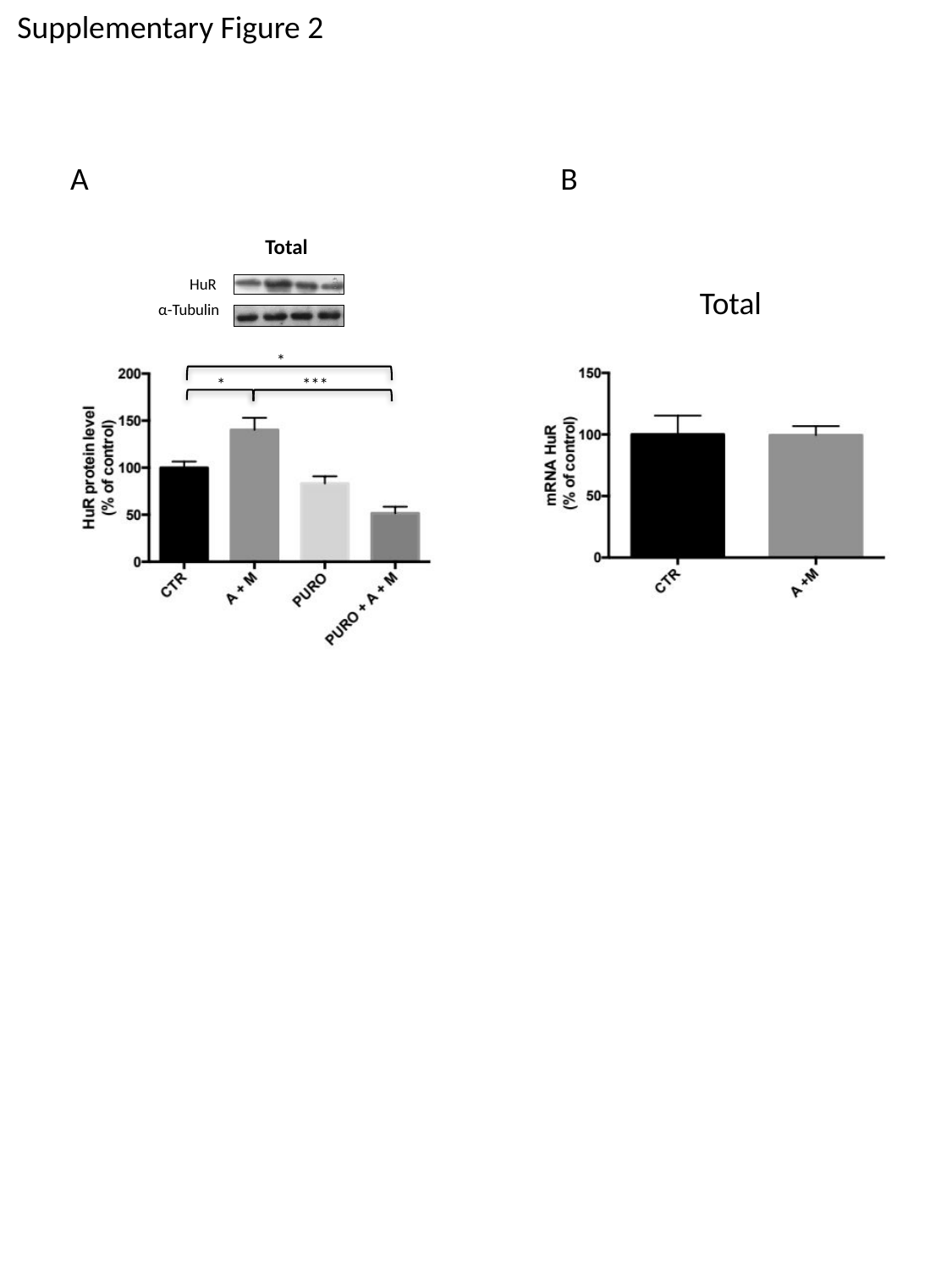

Supplementary Figure 2
A
B
Total
HuR
Total
α-Tubulin
*
*
***

Supplement: Supplementary 2 — Evaluation of HuR protein and mRNA levels following AICAR + MG132 exposure. (A) Representative Western blotting (upper) and densitometric analyses (lower) of HuR protein levels in the total homogenates of ARPE-19 cells exposed to either solvent (CTR) or AICAR + MG132 (A + M) for 2 hrs in the presence or not of puromycin (1 μM, PURO). Optical densities of HuR bands were normalized to α-tubulin, and the results expressed as mean percentages + S.E.M. (n = 3; ∗ p < 0.05; ∗∗∗ p < 0.0001; Tukey's multiple comparison test). (B) Determination by real-time qPCR of HuR mRNA levels in the total homogenate of ARPE-19 cells exposed to either solvent (CTR) or AICAR + MG132 (A + M) for 2 hrs. HuR mRNA levels were normalized in accordance with the corresponding RPL6 mRNA content. The values are expressed as mean percentages + S.E.M. The experiments were performed in duplicate on 4-5 independent sets of cells. [file 4956080.f2.pptx]

## Slide 1
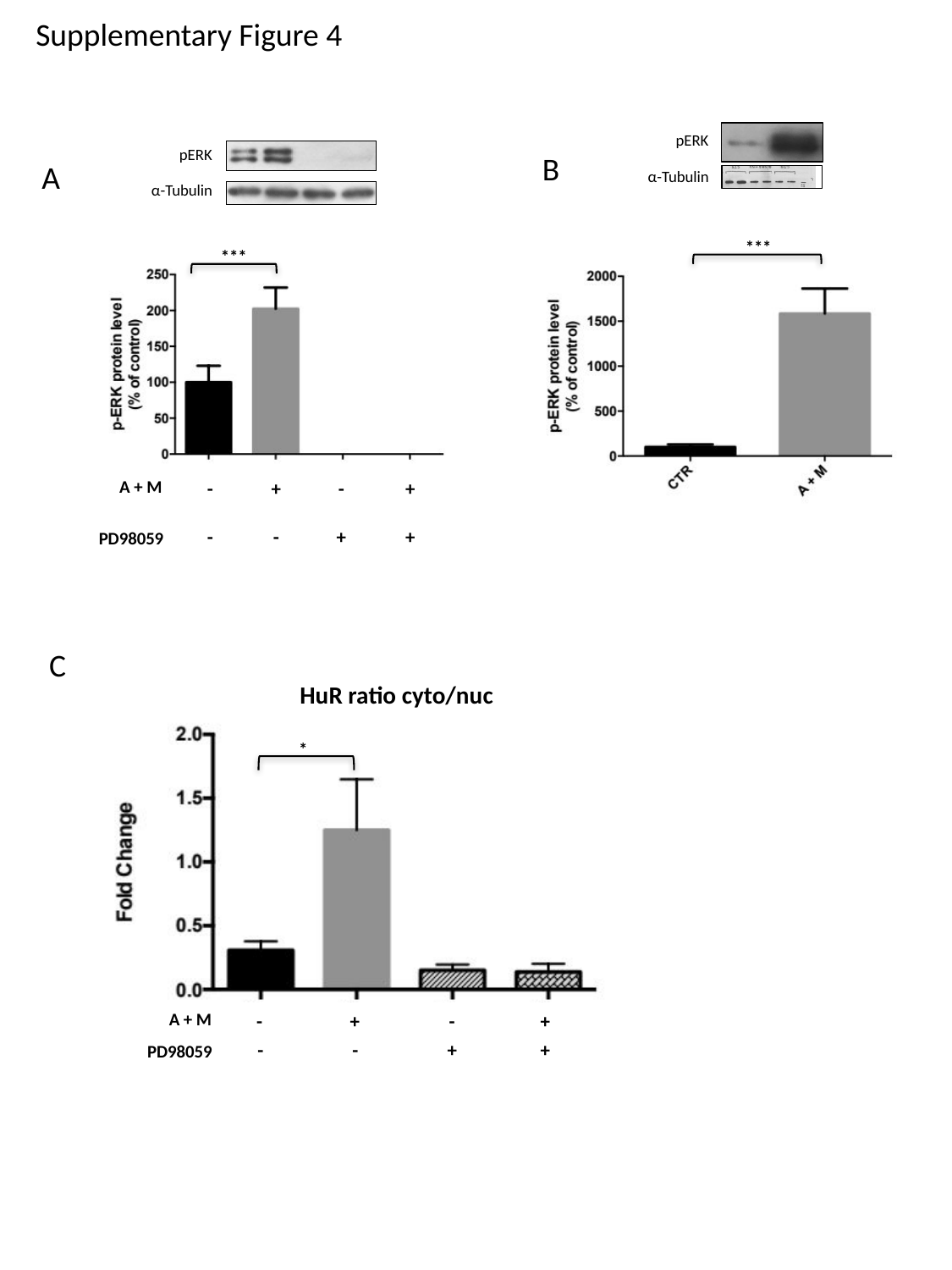

Supplementary Figure 4
pERK
pERK
B
A
α-Tubulin
α-Tubulin
***
***
A + M
-
+
-
+
-
-
+
+
PD98059
C
HuR ratio cyto/nuc
*
*
A + M
-
+
-
+
-
-
+
+
PD98059

Supplement: Supplementary 4 — Effects on Erk1/2 activation following AICAR + MG132 exposure in the presence of Erk1/2 inhibitor. (A) Representative Western blotting (upper) and densitometric analyses (lower) of phospho-Erk1/2 (p-Erk) protein levels in the cytoplasm of ARPE-19 cells exposed to either solvent or AICAR + MG132 (A + M), in the presence or not of Erk1/2 inhibitor (50 μM PD98059) for 2 hrs. Optical densities of p-Erk bands were normalized to α-tubulin, and the results are expressed as mean percentages + S.E.M. (n = 4; ∗∗∗ p < 0.0001; Tukey's multiple comparison test). (B) Representative Western blotting (upper) and densitometric analyses (lower) of phospho-Erk1/2 (p-Erk) protein levels in the cytoplasm of ARPE-19 cells exposed to either solvent or AICAR + MG132 (A + M) for 30 min. Optical densities of p-Erk bands were normalized to α-tubulin, and the results are expressed as mean percentages + S.E.M. (n = 4; ∗∗∗ p < 0.0001; Student's t-tests). (C) The mean ratio + S.E.M. between the cytoplasmic and nuclear signals of HuR protein in the cytoplasm of ARPE-19 cells exposed to either solvent or AICAR + MG132 (A + M), in the presence or not of Erk1/2 inhibitor (50 μM PD98059) for 2 hrs (n = 3; ∗ p < 0.05; Dunnett's multiple comparisons test). [file 4956080.f4.pptx]

## Slide 1
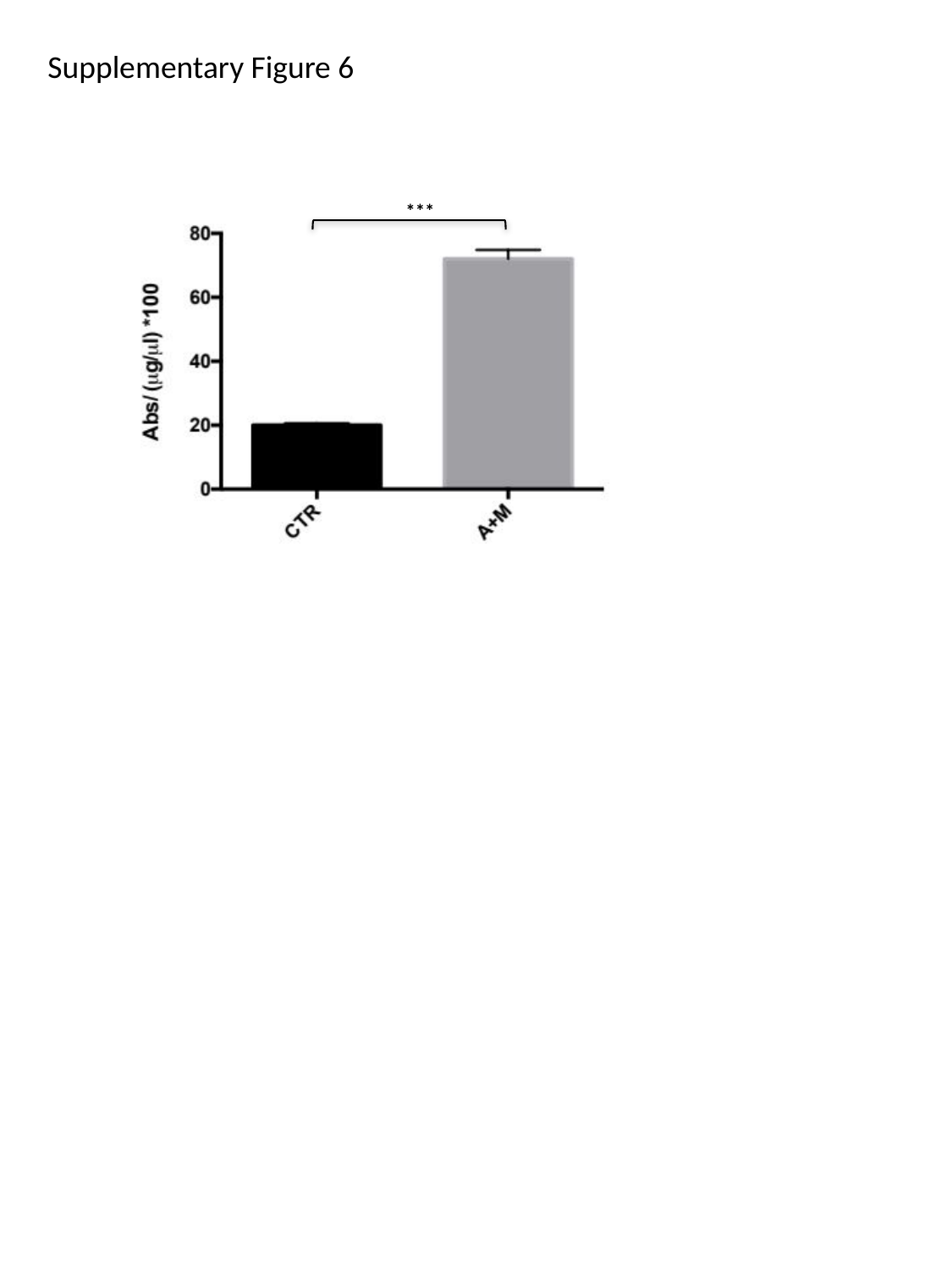

Supplementary Figure 6
***

Supplement: Supplementary 6 — Effects on JNK activation following AICAR + MG132 exposure. Levels of phosphorylated JNK in the cytoplasm of ARPE-19 cells exposed for 2 hrs to either solvent (CTR) or AICAR (A + M) measured by ELISA (n = 4; ∗∗∗ p < 0.0001; Student's t-test). [file 4956080.f6.pptx]

## Slide 1
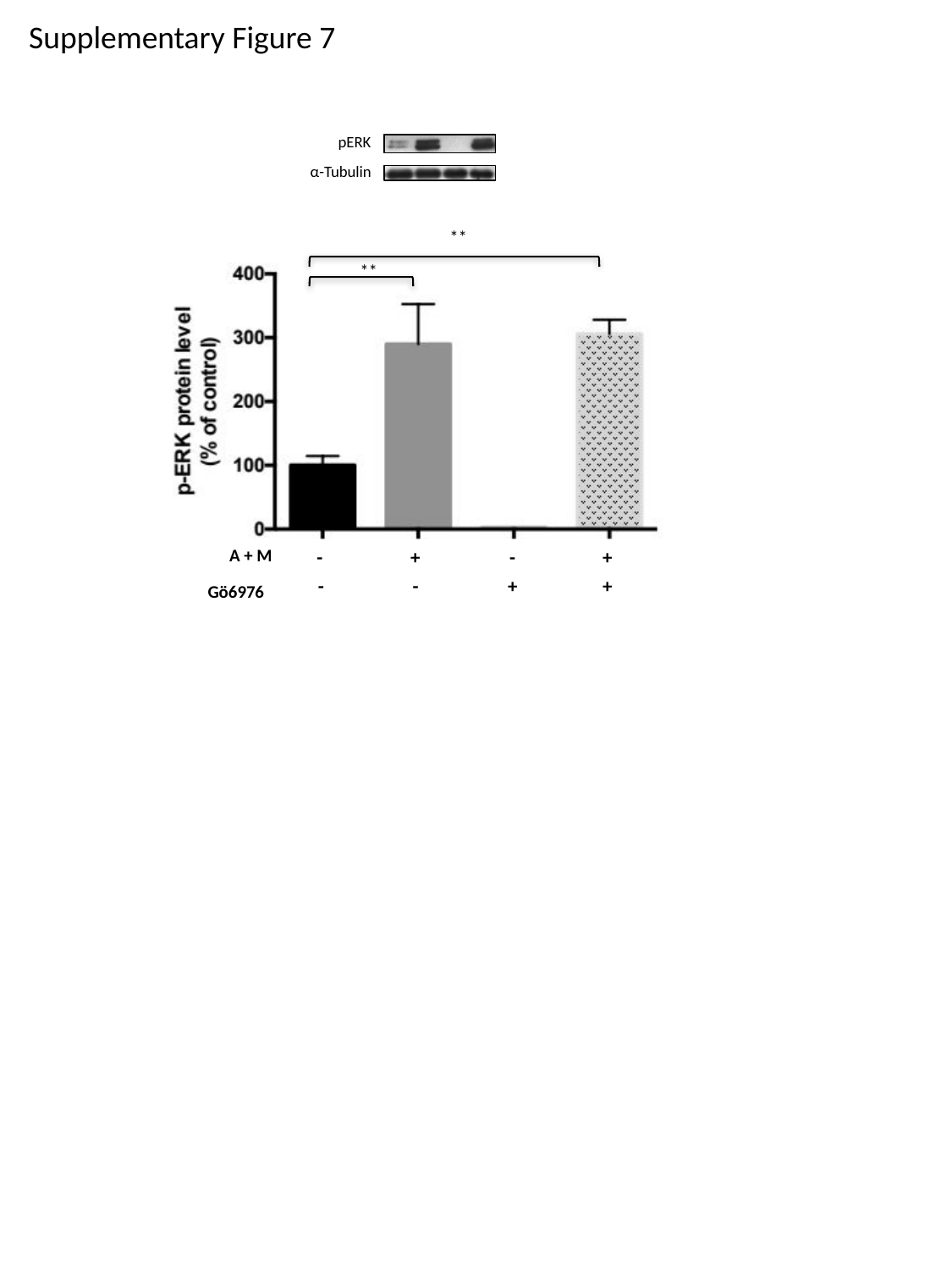

Supplementary Figure 7
pERK
α-Tubulin
**
**
A + M
-
+
-
+
-
-
+
+
Gö6976

Supplement: Supplementary 7 — Effects of cPKC inhibitor on Erk activation. Representative Western blotting (upper) and densitometric analyses (lower) of phospho-Erk1/2 (p-Erk) protein levels in the cytoplasm of ARPE-19 cells exposed to either solvent or AICAR (A + M), in the presence or not of cPKC inhibitor (2 μM Gö6976) for 2 hrs. Optical densities of p-Erk bands were normalized to α-tubulin, and the results are expressed as mean percentages + S.E.M. (∗∗ p < 0.005; n = 4; Tukey's multiple comparison test). [file 4956080.f7.pptx]
