# Supplementary material for: Autophagy Stimulus Promotes Early HuR Protein Activation and p62/SQSTM1 Protein Synthesis in ARPE-19 Cells by Triggering Erk1/2, p38MAPK, and JNK Kinase Pathways
Source: Oxid Med Cell Longev. 2018 Feb 8;2018:4956080. doi: 10.1155/2018/4956080 (PMC5822911; doi:10.1155/2018/4956080)
Supplement: Supplementary 3 — AICAR + MG132 induces de novo translation of HuR mRNA. ARPE-19 cells were treated with either solvent (DMSO) or AICAR + MG132 for 2 hrs followed by polysome separation and Real-time qPCR analysis. HuR transcript level quantification was performed in free RNA, monosomes, and polysome of ARPE-19 cells. Relative expression of HuR was normalized to mRNA of free RNA sample, considering the value of GAPDH as a housekeeping gene. [file 4956080.f3.pptx]

## Slide 1
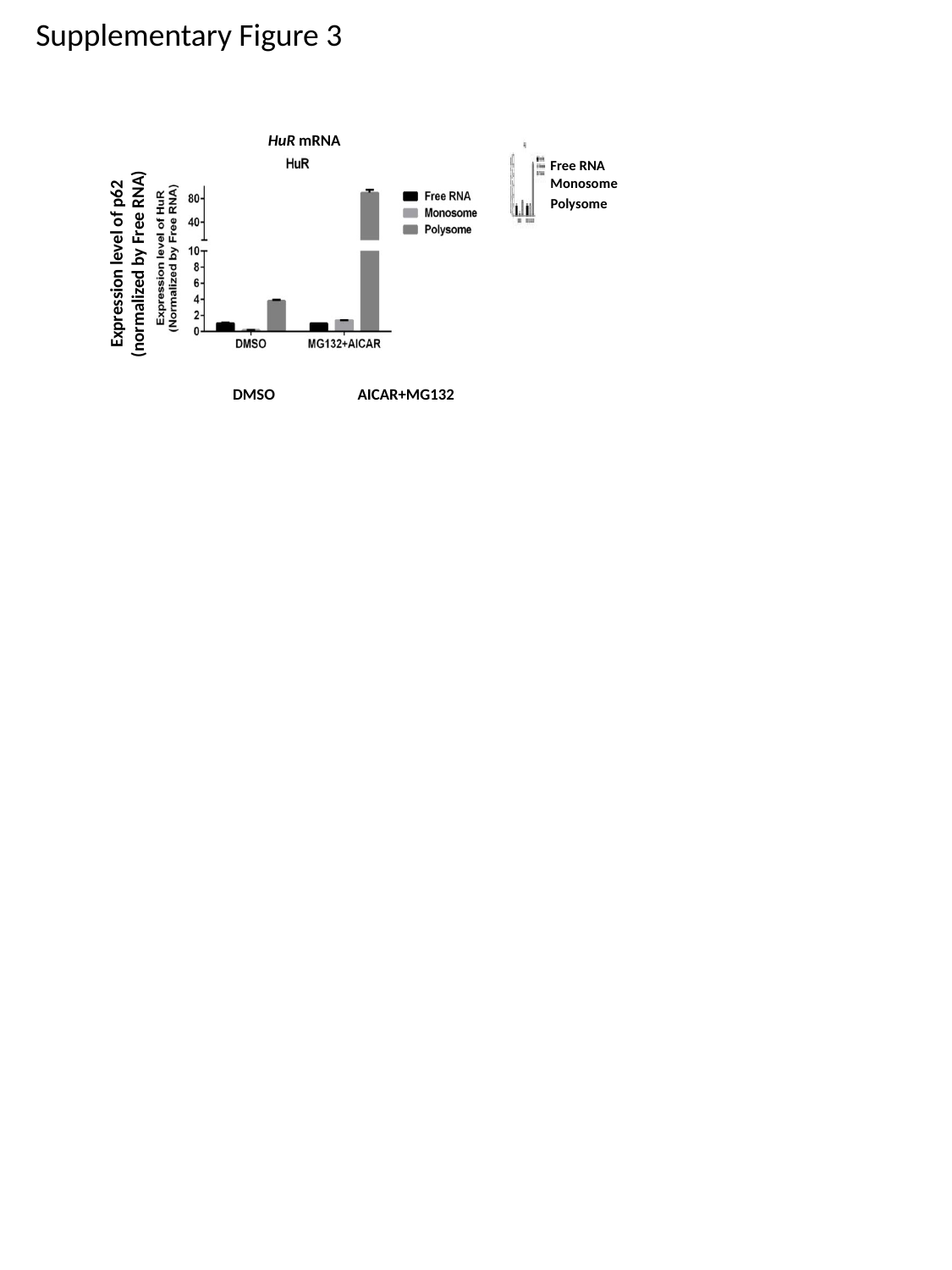

Supplementary Figure 3
HuR mRNA
Free RNA
Monosome
Polysome
Expression level of p62
(normalized by Free RNA)
DMSO AICAR+MG132
