# Supplementary material for: Autophagy Stimulus Promotes Early HuR Protein Activation and p62/SQSTM1 Protein Synthesis in ARPE-19 Cells by Triggering Erk1/2, p38MAPK, and JNK Kinase Pathways
Source: Oxid Med Cell Longev. 2018 Feb 8;2018:4956080. doi: 10.1155/2018/4956080 (PMC5822911; doi:10.1155/2018/4956080)
Supplement: Supplementary 5 — Effects of AMPK inhibitor on HuR translocation. (A) Immunocytochemistry of ARPE-19 cells exposed to either solvent (CTR) or AICAR + MG132 alone or together, in the presence or not of AMPK inhibitor (5 μM CC) for 2 hrs. The left panels of immunocytochemistry show HuR staining (red), the right panels show nuclei staining with DAPI (blue), and the middle panels show merged images. Scale bar: 20 μm. Inserts: immunofluorescence analysis of HuR was performed using a Zeiss Observer Z1 microscope equipped with Apotome module, with a Plan Apochromatic (63x, NA 1.4) objective. Nuclei staining with DAPI (blue). Images were acquired using Zen 1.1 (blue edition) imaging software and assembled with ImageJ software. (B) The ratio between the cytoplasmic and nuclear signals of HuR is calculated as the mean of each ratio in each single cell in every well (n = 3; ∗ p < 0.05 and ∗∗ p < 0.005; Dunnett's multiple comparison test). Imaging (40x magnification) and evaluation of HuR localization were made with the PerkinElmer image plate reader Operetta. [file 4956080.f5.pptx]

## Slide 1
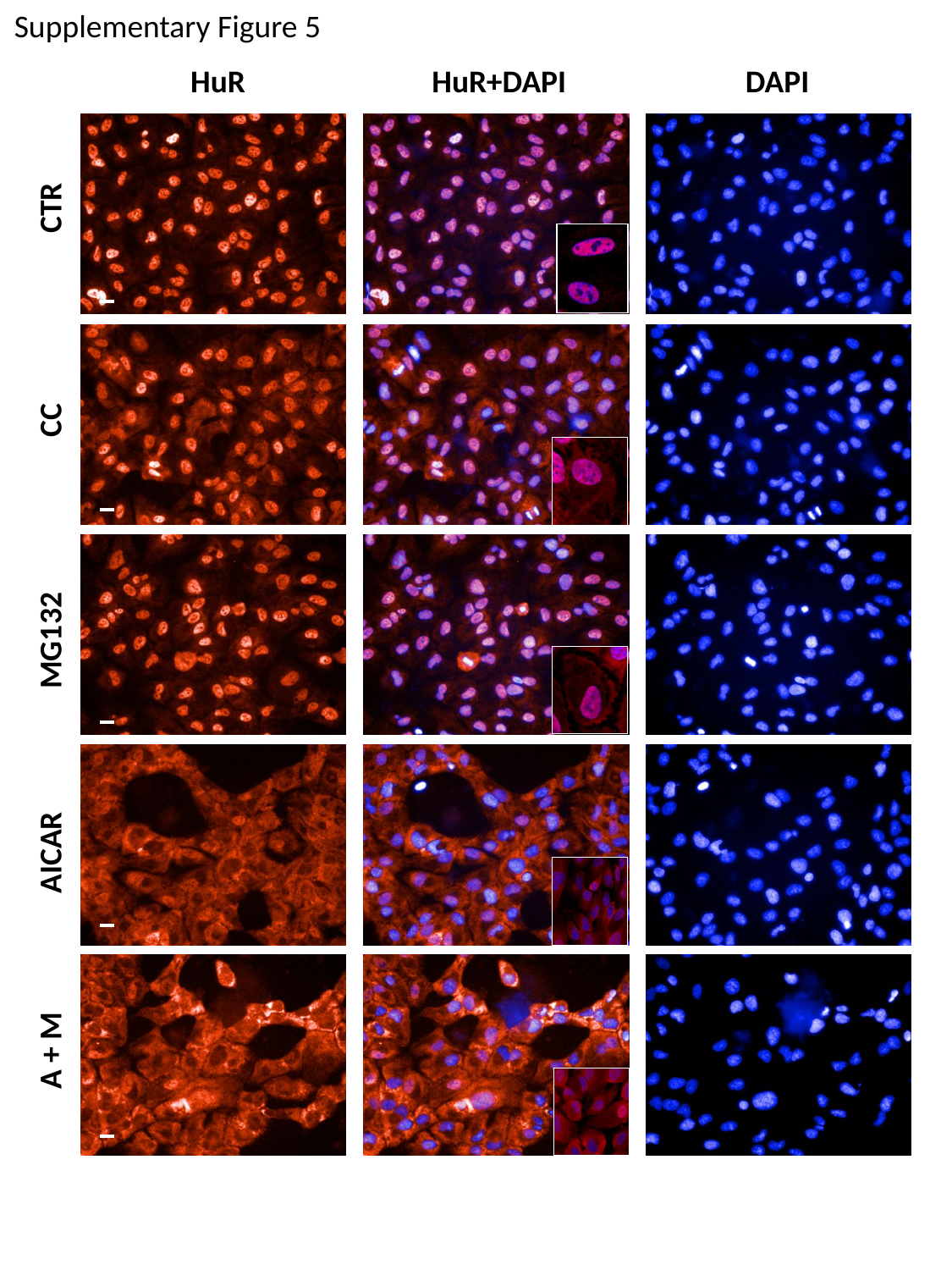

Supplementary Figure 5
HuR HuR+DAPI DAPI
CTR
CC
MG132
AICAR
A + M

## Slide 2
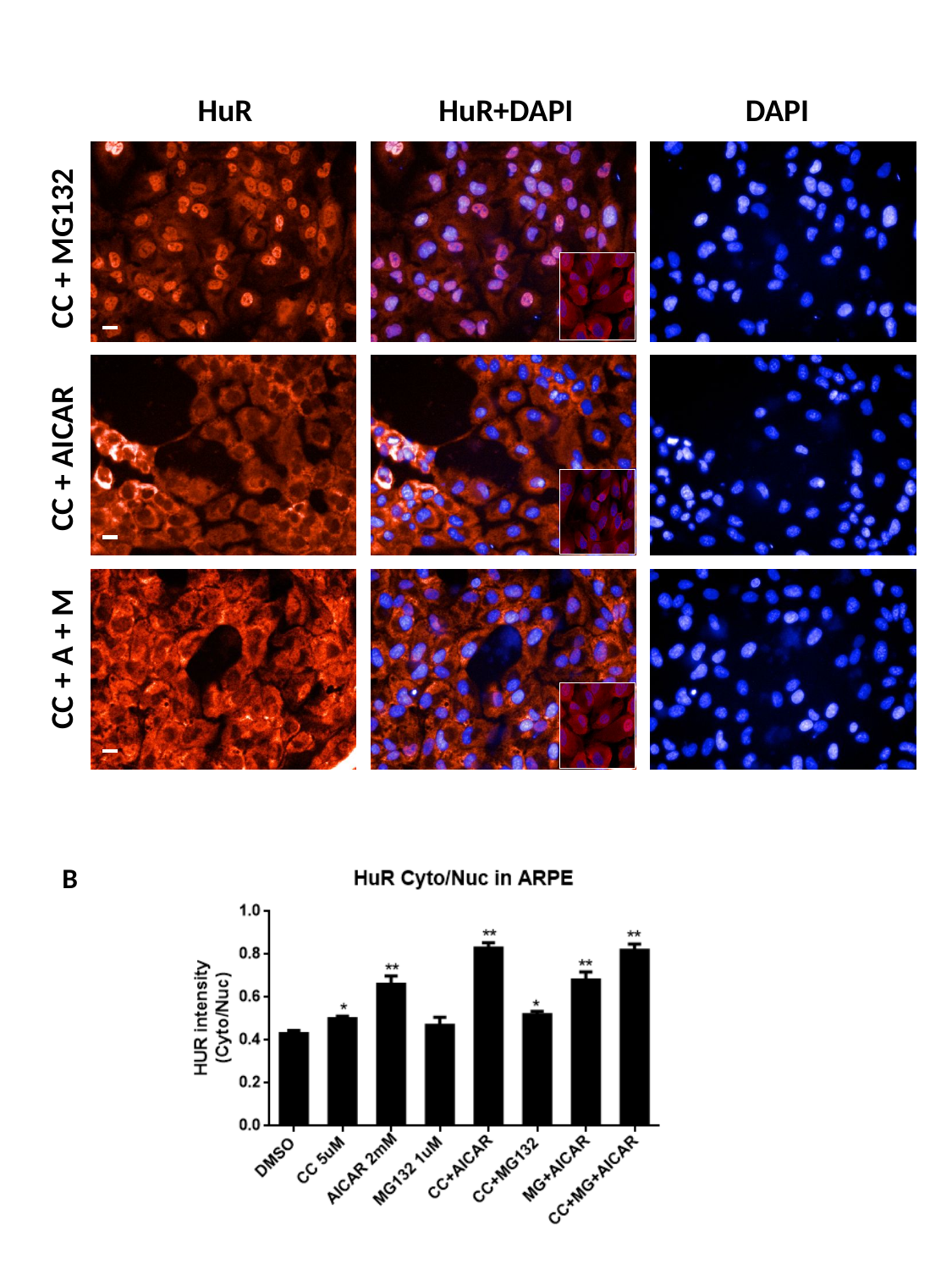

HuR HuR+DAPI DAPI
CC + MG132
CC + AICAR
CC + A + M
B
